# Sensitive Identification of Asymmetries and Neuromuscular Deficits in Lower Limb Function in Early Multiple Sclerosis

**DOI:** 10.1177/15459683241245964

**Published:** 2024-04-13

**Authors:** Anne Geßner, Maximilian Hartmann, Anikó Vágó, Katrin Trentzsch, Dirk Schriefer, Jan Mehrholz, Tjalf Ziemssen

**Affiliations:** 1 Center of Clinical Neuroscience, Neurological Clinic, University Hospital Carl Gustav Carus, TU Dresden, Dresden, Germany; 2 SRH University of Applied Sciences, Gera, Germany; 3 Public Health, TU Dresden, Dresden, Germany

**Keywords:** multiple sclerosis, asymmetries, neuromuscular deficits, single-leg countermovement jump, lower limb assessment

## Abstract

**Background:**

In the early stages of multiple sclerosis (MS), there are no objective sensitive functional assessments to identify and quantify early subclinical neuromuscular deficits and lower limb strength asymmetries during complex movements. Single-countermovement jumps (SLCMJ), a maximum single leg vertical jump, on a force plate allow functional evaluation of unilateral lower limb performance in performance diagnostics and could therefore provide early results on asymmetries in MS.

**Objective:**

Objective evaluation of early lower limb neuromuscular deficits and asymmetries in people with multiple sclerosis (pwMS) using SLCMJ on a force plate.

**Methods:**

A study was conducted with pwMS (N = 126) and healthy controls (N = 97). All participants performed 3 maximal SLCMJs on a force plate. Temporal, kinetic, and power jump parameters were collected. The Expanded Disability Status Scale (EDSS) was performed on all participants. A repeated measures analysis of covariance (ANCOVA) with age, Body-Mass-Index, and gender as covariates was used.

**Results:**

PwMS with normal muscle strength according to the manual muscle tests showed significantly reduced SLCMJ performance compared to HC. In both groups, jumping performance differed significantly between the dominant and non-dominant leg, with higher effect size for pwMS. A significant interaction effect between leg dominance and group was found for propulsive time, where the pwMS showed an even higher difference between the dominant and non-dominant leg compared to HC. Furthermore, there was a significant small correlation between leg asymmetries and EDSS in pwMS.

**Conclusion:**

The study shows that the SLCMJ on a force plate is suitable for the early detection of subclinical lower limb neuromuscular deficits and strength asymmetries in MS.

## Introduction

Multiple sclerosis (MS) is a chronic multifocal inflammatory and neurodegenerative disease of the central nervous system causing variable functional deficits in different neurological functional systems (eg, motor and sensory function and coordination).^1^ Early symptoms are neuromuscular deficits including a decrease in muscle strength, which are associated with impairment in activities of daily living and quality of life.^2^ Muscle strength, especially in the lower limb, is more frequently impaired in people with multiple sclerosis (pwMS) compared to healthy controls (HC) already at early disease stage (reduction of ~25%). In addition, pwMS show reduced muscle activation and increased muscle fatigue.^3^ These neuromuscular deficits can be frequently demonstrated by muscle strength asymmetries.^4,5^ Strength asymmetries are defined as a relative difference of more than 10% to 15% in maximal strength between opposing muscle groups.^6^ Workman et al indicated that muscle strength asymmetry in pwMS is associated with increased muscle energy expenditure, early fatigue and impaired gait, and balance compared with HC.^7^ Evidence is mainly based on lower limb strength asymmetries in pwMS with mild to moderate disability (Expanded Disability Status Scale [EDSS] < 6.5) and has found that asymmetries proceed with increasing disability.^8^ However, to date, no studies have investigated muscle strength asymmetries at already early stage of MS when the gold standard of disability assessment, the EDSS could not demonstrate muscle strength deficits.^9^ Identifying, specifying, and monitoring subtle neuromuscular impairments is crucial for optimal disease-modifying and symptomatic treatment in individualized innovative neurorehabilitation management of MS at an early stage.^10,11^

Methods used to detect early neuromuscular deficits and lower limb strength asymmetries include manual muscle function tests (MMT) and isometric or isokinetic dynamometer measurements.^12,13^ The MMT are performed as part of the EDSS in the pyramidal functional system and are scored on the British Medical Research Council (BMRC) scale from 0 (when no muscle contraction is detected) to 5 (if there is normal muscle strength) for both legs and the different muscle groups (hip flexors, knee flexors, knee extensors, plantar flexion, and dorsiflexion).^14,15^ Although MMT are simple and does not require technical equipment, there are some limitations in its application and interpretation.^16^ Limitations of the MMF include the need for extensive training and experience on the part of the examiner and their subjective interpretation, the assessment of isolated concentric muscle strength only, and disregard for functional assessment of the entire lower limb muscle chain.^17,18^ To date, there are no objectively sensitive functional assessments to identify and quantify early subclinical neuromuscular deficits and lower limb strength asymmetries during complex movements.^19,20^ Several studies indicate that objective assessments with higher levels of challenge and complexity are better at identifying subtle deficiencies.^19,21,22^ Two pilot studies of Kirkland et al provide initial evidence that jumping, as a complex and innovative novel assessment tool in MS, can reveal differences between pwMS with mild disability and HCs, and is potentially useful for assessing lower limb neuromuscular function in pwMS.^21,23^ In our previous pivotal study, we found that pwMS without any strength, coordination, and sensory impairments of the legs required more complex assessments, such as the countermovement jump (CMJ), a bipedal vertical jump, to identify neuromuscular deficits below the clinical threshold of EDSS.^24^ The pwMS with full motor function showed significantly reduced CMJ performance in almost all observed kinetic, temporal, and performance parameters compared to the HC in this study. With increasing disability in pwMS, it was also found that jump performance decreased signiﬁcantly. The results of the studies by Kirkland et al and our previous study are consistent in that the domains of coordination, balance, proprioception, and strength can be assessed with jumps in 1 assessment and identify subtle specific neuromuscular deficits in MS.^24,25^

In addition to the CMJ, there are a variety of jump tests to assess unilateral lower limb strength asymmetry.^26^ The Single leg countermovement jump (SLCMJ) is one of the most sensitive tests for unilateral neuromuscular function and most used in sports performance diagnostics.^26^ A variety of studies confirm the high validity and reliability of the SLCMJ on a force plate for the objective assessment of the symmetry and neuromuscular function.^27,28^

In patients with cerebral palsy, the SLCMJ is suitable for measuring jump height and power generation, thus providing additional information to the usual maximal force measurements.^29^ The SLCMJ is a high-challenge task for the neuromuscular system, with greater demands on the limb regarding to balance, control, coordination, and strength than bipedal jumps.^30^

In addition, the SLCMJ works on the principle of the stretch-shortening cycle (high-intensity eccentric contraction immediately before a rapid concentric contraction), which occurs in natural movements such as walking and running.^30,31^ Therefore, the SLCMJ could simulate complex unilateral natural movements of activities of daily living (eg, climbing stairs) and identify neuromuscular asymmetries in MS. To our knowledge, no studies have investigated the performance of the SLCMJ on a force plate in MS.

The first objective of this study was therefore (i) to investigate the SLCMJ performance between pwMS and HC and second (ii) to evaluate the suitability of the SLCMJ to detect subclinical early neuromuscular deficits and lower limb asymmetries in pwMS using SLCMJ on a force plate. The third objective (iii) was to assess the relationship between EDSS and symmetry in SLCMJ performance in pwMS.

## Materials and Methods

### Participants

We conducted a prospective cross-sectional study at our specialised MS Center at the Center of Clinical Neuroscience of the Department of Neurology, University Hospital Carl Gustav Carus, Dresden, Germany. Between April 2022 and February 2023 we screened 213 subjects (79 HC without neurological disease and 134 pwMS) admitted to our department. We recruited patients who met the following criteria:

Inclusion criteria: (1) conﬁrmed diagnosis of MS according to McDonald criteria, (2) EDSS Score between 0 and 5.0, (3) age between 18 and 50 years and (4) written informed consent, and (5) the ability to walk >500 m without an aid. Exclusion criteria were the presence of any orthopedic or surgical condition affecting jumping ability. Insecurity during jumping and pregnancy were also excluded.

The study was approved by the local ethics committee (BO-EK-320062021) and all participants provided written informed consent for the study.

### Measures and Procedures

#### Expanded Disability Status Scale

To examine the clinical status, the EDSS was assessed using neurostatus by certiﬁed raters in pwMS, including MS-speciﬁc pyramidal, cerebellar, and sensory Functional System Score (FSS) in all participants.^14^ The EDSS is the most used disability scale in MS and is well established among neurologists.^32^ In this study, HC were also examined with the complete EDSS.

As part of the EDSS in the pyramidal FSS, MMT were assessed using the BMRC for the hip flexors, knee flexors, knee extensors, plantar flexors, and dorsal extensors for both legs to determine lower limb muscle strength.^18^ The MMT provides the following grades: 0 = paralysis; 1 = only a trace or flicker of muscle contraction is seen or felt; 2 = muscle movement is possible with gravity eliminated; 3 = muscle movement is possible against gravity; 4 = muscle strength is reduced but the movement against resistance is possible; and 5 = normal strength.^18^ Participants were classified according to MMT as follows:

(a) Participants with normal muscle strength: full strength, grade 5 in all assessed muscle groups of the lower limb
(b) Participants with reduced muscle strength: not full strength, grade 4, or less in 1 or more muscle groups of the lower limb

#### Single Leg Countermovement Jump

**Figure 1.**
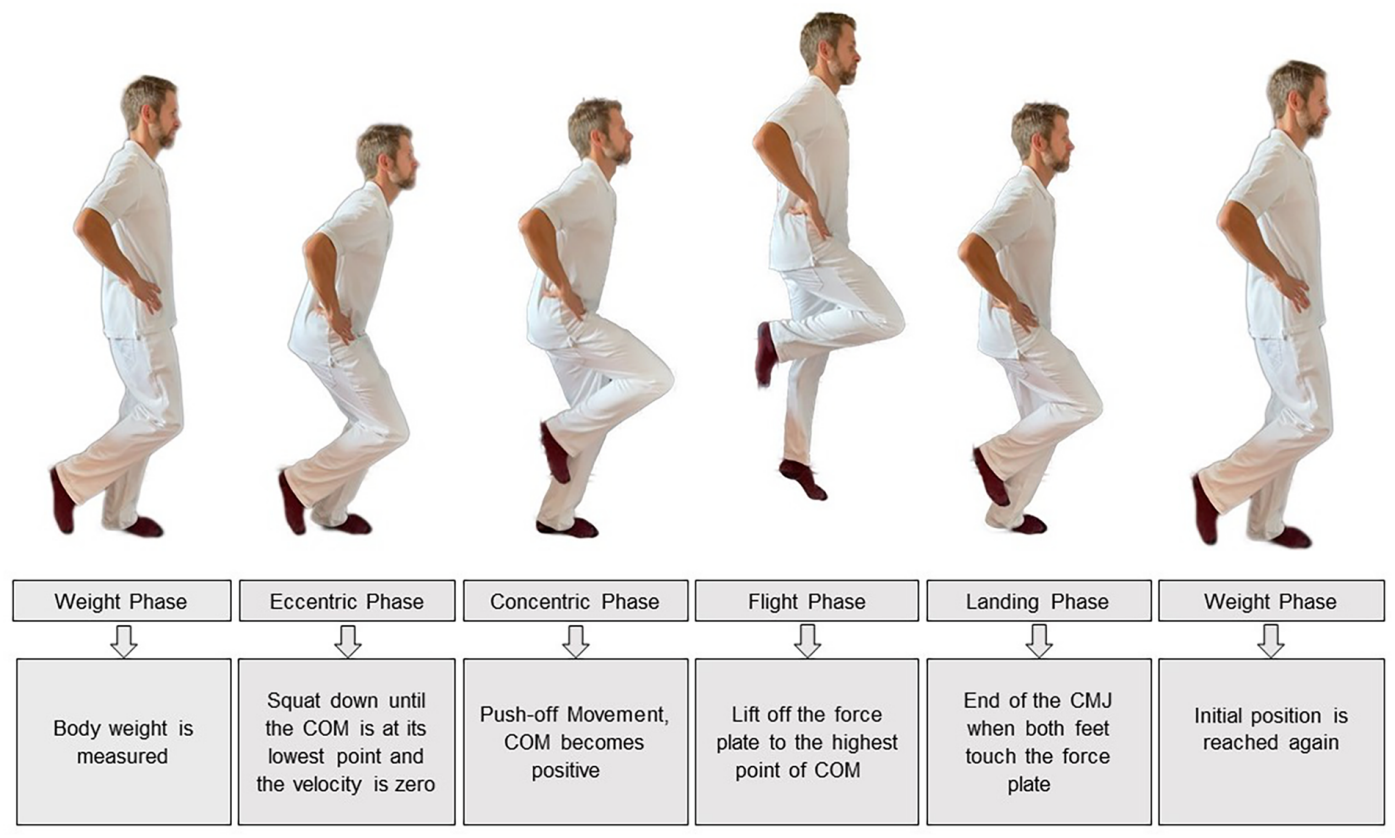
Single leg countermovement jump phases. The SLCMJ were performed on a force plate and can be divided into 5 phases: weight phase, eccentric phase, concentric phase, flight phase, and landing phase. First, the patient stands still with extended knee and hips on the force plate and the body weight is measured. The patient then begins a short countermovement on 1 leg with hip and knee flexion in which the body weight is reduced below a threshold of 5% and the eccentric phase begins. The eccentric phase is characterised by flexion of the hip, knee, and ankle of the jumping leg until the center of mass (COM) is at its lowest and the speed is zero. This is followed by the concentric phase, which is characterised by a forceful extension of the hip, knee, and ankle to move the COM upwards and push off the force plate. The time from take-off from the force plate to the highest point of the COM is called the flight phase. The SLCMJ ends with the landing phase when both feet touch the force plate and the starting position is reached.

All participants performed 3 maximal SLCMJs (right and left) without arm swing on a force plate. Prior to the jumps, a physiotherapist explained and demonstrated the jumping technique to each participant (see Figure 1). Participants were instructed to jump as high as possible on 1 leg and to keep the jumping leg extended during the flight phase of the jump. Standardised jumps were initiated with the right leg, and there was a 5-second interval between each of the 3 SLCMJs. After performing 3 jumps on their right leg, the participants had a rest of 1 to 2 minutes before the same testing procedure was conducted on their left leg. Any SLCMJs performed inadvertently with an arm swing were excluded from the study. Prior to data collection, all participants practised the jump tests once on each side. The jump tests were carried out using everyday clothing and socks.

#### Data Collection of the Jump Parameter

Three-dimensional ground reaction forces (*Fx*, *Fy*, and *Fz*) and force moments (*Mx*, *My*, and *Mz*) during the SLCMJ were recorded at a frequency of 1000 Hz using a portable single force plate manufactured by Advanced Mechanical Technology Inc., Watertown, MA, USA, AccuPower-O. Force plates are seen as the gold standard and a valid method for measuring vertical jump performance.^33,34^ A dedicated biomechanical analysis software (AccuPower Solutions, version 1.5.4.2082. Watertown, MA, USA) was used to analyze the jump parameters from the recorded ground reaction forces of the force plate.

As a function of the different jump phases, the most common and reliable jump parameters in sports medicine have been selected.^35-37^ In addition, parameters already used in the CMJ to detect differences between pwMS without motor disability and HC were analyzed.^24^ The jump parameters were divided into temporal, kinetic, and performance parameters.

In addition to this classification of the parameters, jump parameters of the eccentric and concentric phase as well as flight phase were considered. This provides a comprehensive overview of the complete muscle function of the lower limb. Table 1 shows a description of the recorded jump parameters.

**Table 1.** Jump Parameters Measured by Force Plate.

	Jump parameters	Description	Interpretation
Temporal	Flight time (s)^d^	Time in the air from jump take-off to landing	Longer = “better”
	Braking time (s)^b^	Duration of the eccentric phase	Shorter = “better”
	Propulsive time (s)^c^	Duration of the concentric phase	Shorter = “better”
Kinectic	Average negative power (W/kg)^b^	Average power during eccentric phase	Higher = “better”
	Average positive power (W/kg)^c^	Average power during concentric phase	Higher = ”better”
	Force at zero velocity (N/kg)^b^	Maximum force during eccentric phase	Higher = “better”
	Peak force (N/kg)^c^	Maximum force during concentric phase	Higher = “better”
	BPIR^f^	Ratio of braking to propulsive impulse	Lower = “better”
Performance	FTCTR^d+f^	Ratio of ﬂight to contraction time	Higher = “better”
	Jump height (cm)^d^	Jump height calculated by force impact	Higher = “better”

Abbreviations: BPIR, Brake to Propulsive Impulse Ratio; FTCTR, ﬂight time to contraction time ratio.

Phases: ^a^Weight phase; ^b^Eccentric phase. ^c^Concentric phase. ^d^Flight phase. ^e^Landing phase. ^f^Contraction phase (eccentric and concentric phase).

### Statistical Analysis

Statistical analyses were performed using IBM Statistical Package for the Social Sciences for Windows, version 28 (IBM Corp, Armonk, NY, USA). Subjects who performed 3 jumps each on the right and left side were included in the statistical analysis. For statistical analysis, the right and left legs were divided into dominant and non-dominant legs. The dominant leg was defined as the leg with the greater jump height.^26^ Jump height is often used as an indicator for muscle power and many studies have shown a positive relationship between lower limb power and jump performance.^27,38-40,41^

The distribution of all jump parameters was visually checked via histograms and *Q–Q* plots and the assessment of normality was supplemented with the Shapiro–Wilk test. Jump variables were log transformed before analyses to stabilize variance and to optimize normality for (slightly) right-skewed distributions of jump outcomes. Continuous data are presented as mean ± standard deviations or medians ± interquartile range, as applicable. Absolute numbers and percentages were used to describe categorical variables. The Limb Symmetry Index (LSI) was used to calculate the percentage difference in the contralateral muscle strength of the lower limb. A difference >15% was defined as asymmetry between the contralateral muscle groups. The LSI was calculated as follows^6^:

LSI=(non−dominantleg/dominantleg)*100

An ANCOVA with repeated measures (rmANCOVA) and the covariates age, Body-Mass-Index (BMI), and gender was used to assess the effect of leg dominance on jumping performance in pwMS with normal muscle strength (pwMS-NM) and HC. The defined factors included in this analysis were the within-subject factor “leg dominance” (dominant and non-dominant leg) and the between-subject factor “group” (pwMS-NM and HC) as well as the interaction of both factors (leg dominance × group). Necessary assumptions for performing the selected models were checked, such that a linear relationship between covariables and the dependent variable (jump outcomes) was confirmed using scatterplots. Homogeneity of regression slopes was confirmed before conducting the analysis. Mauchly’s test of sphericity did not indicate a violation of the assumption of sphericity as only 2 levels of the within-subject factor (leg dominance) occurred. After identifying the main effects of the assessed factors and their interaction, Bonferroni-based post-hoc comparisons were performed to determine the simple (main) effects of the interaction term. Appropriate adjusted *P* values and effect sizes were reported. In all models, partial eta squares (ηp^2^) as a measure of effect size were interpreted as small (ηp^2^ > .01), medium (ηp^2^ > .06), or large (ηp^2^ > .14).^42^

## Results

### Participants

**Figure 2.**
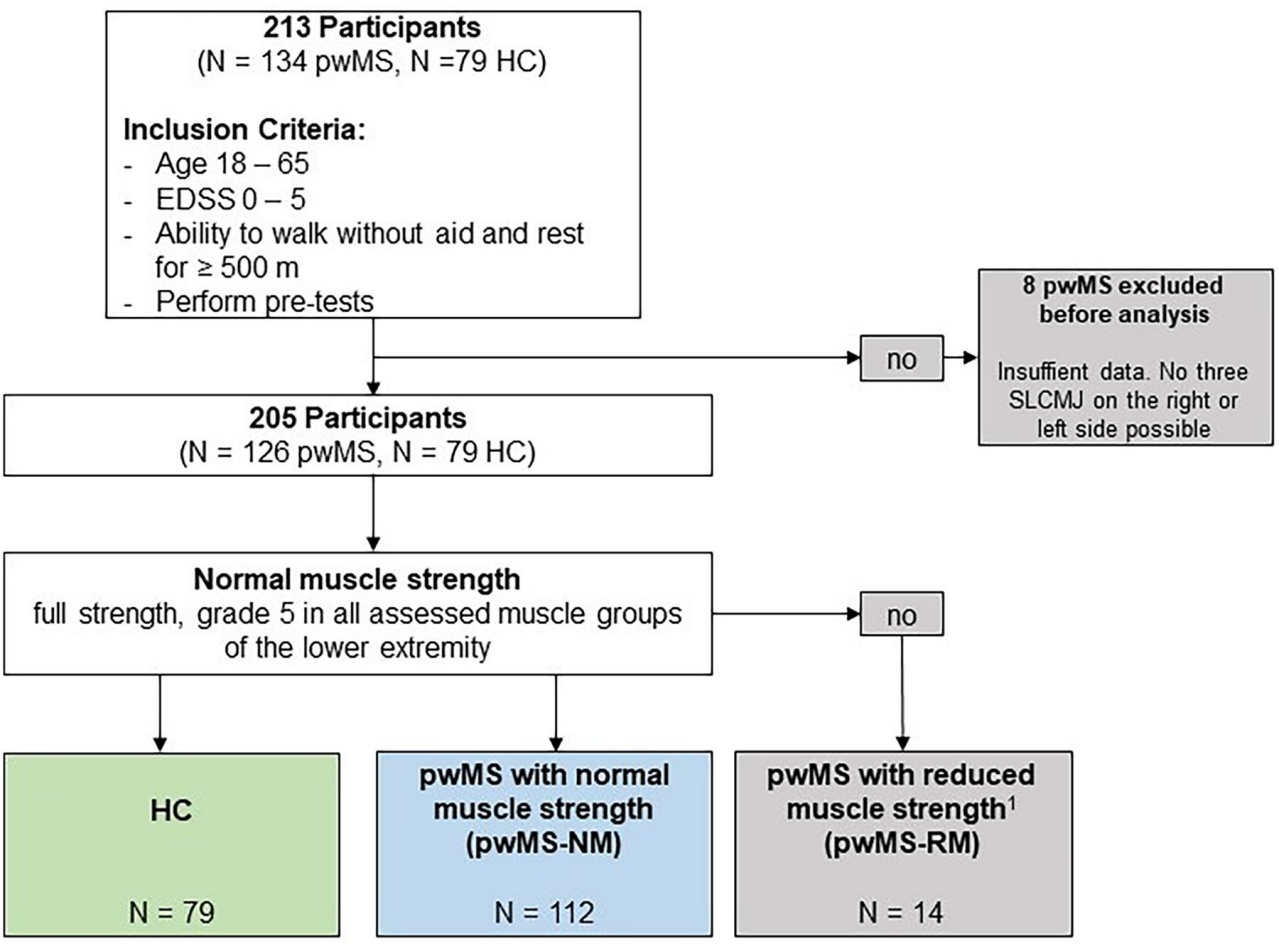
Flowchart of the study population. A total of 205 participants were included in the statistical analysis. Further, for comparability in terms of muscle strength between pwMS and HC, 2 MS groups were divided in pwMS with normal muscle strength (pwMS-NM) and pwMS—with reduce muscle strength (pwMS-RM). PwMS-NM were deﬁned as having normal muscle strength according to MMT. In this item, the pwMS-NM were equal to the HC. The pwMS-NM and HC did not differ significantly by age, gender, or BMI. The low median for pyramidal FS (pwMS-NM: median = 1, IQR = 1-1) and cerebellar FS (pwMS-NM: median = 0, IQR = 0-1) suggest little to low motor function impairment in pwMS-NM. 1 = not full strength, grade 4, or less in 1 or more muscle groups of the lower limb. Abbreviations: EDSS, Expanded Disability Status Scale; pwMS, people with multiple sclerosis; HC, healthy controls; pre-tests, heel stand, toe stand, and squats.

A total of 205 participants (126 pwMS and 79 HC) were included in the statistical analysis (see Figure 2). The characteristics of the participants are summarized in Table 2. No significant differences in age, gender, and BMI were observed between pwMS and HC. The HC showed no reduction in lower limb muscle strength. A total of 14 pwMS showed a reduction in muscle strength (pwMS-RM) and 112 pwMS showed normal muscle strength (pwMS-NM).

**Table 2.** Characteristics of the Study Population (N = 205).

		pwMS (N = 126)	HC (N = 79)	*P*-value
Age (y)		36.67 (±8.49)	37.86 (±10.94)	.770
Females N (%)		84 (66.7)	48 (60.1)	.390
BMI		24.76 (±4.63)	24.21 (±3.36)	.821
Disease duration (years)		7.24 (±5.14)	n.a	—
Muscle strength according to MMT lower limb N (%)				
NM		112 (88.9)	79 (100)	<.001
RM		14 (11.1)	—	
MS Type				
	RRMS	100%	n.a	—
EDSS	Median (IQR)	1.5 (1.5-2.5)	1.0 (0-1.5)	<.001
	Pyramidal FSS	1.0 (1.0-1.0)	0 (0-1.0)	<.001
	Cerebellar FSS	0 (0-1.0)	0 (0-0)	<.001
	Sensory FSS	0 (0-1.0)	0 (0-0)	<.001

Abbreviations: pwMS, people with Multiple Sclerosis; HC, Healthy Controls; BMI, Body-Mass-Index; MMT, Manual Muscle Tests; NM, normal muscle strength; RM, reduce muscle strength; MS, Multiple Sclerosis; RRMS, Relapsing-Remitting Multiple Sclerosis; EDSS, Expanded Disability Status Scale; FSS, Functional System Score; IQR, Interquartile Range.

Data presented as mean (±standard deviation) unless speciﬁed otherwise.

### Descriptive Analysis in SLCMJ Performance in pwMS and HC

The characteristic of the SLCMJ performance of the dominant and non-dominant leg for pwMS and HC is shown in Table 3. The dominant leg showed a better jump performance (dominant leg > non-dominant leg) for all jump parameters in both groups. Overall, the pwMS showed reduced SLCMJ performance on both the dominant and non-dominant leg compared to the HC. The difference between the dominant and non-dominant leg for all jump parameters was 14.2% for pwMS and 11.9% for HC. A higher difference in leg dominance was observed in pwMS-RM (18.4%) than in pwMS-NM (13.7%). In all groups an asymmetry was observed in the jump parameters of the eccentric phase (braking time and negative power).

**Table 3.** Jump Parameters in pwMS and HC.

	pwMS all (N = 126)			pwMS-RM (N = 14)			pwMS-NM (N = 112)			HC (N = 79)		
Parameter	ND	D	LSI (%)	ND	D	LSI (%)	ND	D	LSI (%)	ND	D	LSI (%)
Flight time (s)	0.18 ± 0.04	0.22 ± 0.09	11.9	0.15 ± 0.03	0.27 ± 0.20	25.4	0.19 ± 0.04	0.22 ± 0.07	10.2	0.21 ± 0.04	0.24 ± 0.08	10.3
Braking time (s)	0.35 ± 0.20	0.22 ± 0.14	28.9	0.34 ± 0.18	0.21 ± 0.10	30.6	0.36 ± 0.21	0.23 ± 0.15	28.62	0.29 ± 0.19	0.20 ± 0.11	23.8
Propulsive time (s)	0.40 ± 0.15	0.32 ± 0.09	15.9	0.41 ± 0.14	0.36 ± 0.11	10.8	0.41 ± 0.16	0.32 ± 0.08	16.5	0.36 ± 1.0	0.32 ± 0.07	12
FZV (N/kg)	13.74 ± 1.65	14.80 ± 1.86	7	13.19 ± 1.78	14.26 ± 2.33	7.1	13.80 ± 1.62	14.87 ± 1.79	7	14.41 ± 1.73	15.42 ± 1.83	4.1
Peak force (N/kg)	16.27 ± 1.49	17.02 ± 1.72	4.3	16.40 ± 1.60	17.29 ± 1.86	5	16.25 ± 1.7	16.98 ± 1.5	4.3	16.17 ± 1.59	16.89 ± 1.79	6.4
Negative power (W/kg)	−1.63 ± 0.72	−2.02 ± 0.75	20.4	−1.09 ± 0.54	−1.47 ± 0.71	25.1	−1.7 ± 0.71	−2.09 ± 0.73	19.76	−2.2 ± 0.92	−2.59 ± 0.91	15.8
Positive power (W/kg)	7.94 ± 1.81	8.81 ± 1.81	10.1	6.89 ± 1.49	7.99 ± 6.89	13.8	8.07 ± 1.80	8.91 ± 1.85	9.6	8.56 ± 1.82	9.33 ± 1.94	8.1
Jump height (cm)	4.27 ± 2.16	5.21 ± 2.84	14.5	2.70 ± 1.1	3.59 ± 1.1	24.5	4.47 ± 1.80	5.19 ± 2.11	13.3	5.51 ± 1.15	6.39 ± 2.31	14.2
FTCTR	0.25 ± 0.07	0.31 ± 0.09	15.2	0.22 ± 0.07	0.30 ± 0.08	23.5	0.26 ± 0.07	0.31 ± 0.09	14.1	0.28 ±0.08	0.32 ± 0.09	12

Abbreviations: pwMS, people with Multiple Sclerosis, pwMS-RM, people with multiple sclerosis with reduce muscle strength, pwMS-NM, people with multiple sclerosis with normal muscle strength, HC, healthy controls, ND, non-dominant leg, D, dominant leg, LSI, Limb-Symmetry-Index, FZV, Force at Zero Velocity, FTCTR, Flight-Time–Contraction-Time Ratio.

The LSI > 15% is defined as asymmetry. Data presented as mean (±standard deviation) unless speciﬁed.

### SLCMJ Performance in pwMS-NM and HC

**Figure 3.**
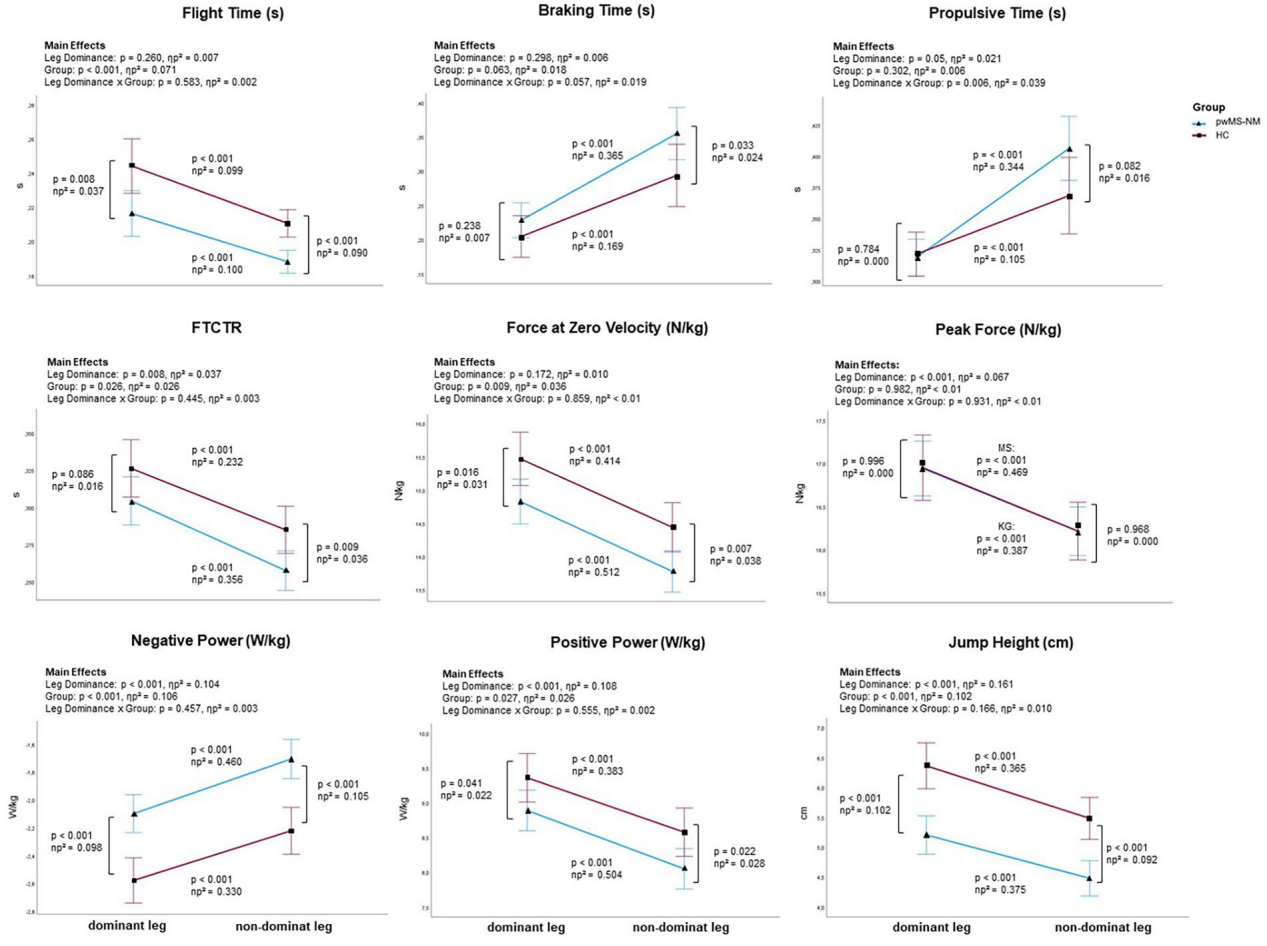
Graphical overview of the rmANCOVA main effects and Bonferroni-based post-hoc comparisons adjusted for age, gender, and BMI. A significant interaction effect was only found for propulsive time. Nevertheless, the pairwise comparisons for all parameters are shown in this figure to answer research objective I (to investigate the SLCMJ performance between pwMS and HC) and II (to evaluate the suitability of the SLCMJ for detecting subclinical early neuromuscular deficits and lower limb asymmetries in pwMS using SLCMJ on a force plate).

In the rmANCOVA analysis, only study participants with normal lower limb muscle strength in both legs according to the MMT tests were included, to assess the suitability of the SLCMJ for detecting subclinical early neuromuscular deficits and asymmetries (pwMS-NM = 112, HC = 79). A significant main effect of leg dominance (dominant and non-dominant) for all kinetic parameter (except force at zero velocity), all performance parameter and propulsive time with the highest effect sizes for jump height (ηp^2^ = .161) followed by positive power (ηp^2^ = .108) and negative power (ηp^2^ = .104) were observed (see Figure 3). A significant group effect (pwMS-NM and HC) was found for all performance parameters, flight time, force at zero velocity, negative power, and positive power. The highest effect sizes of the group effect, similar to the effect of leg dominance were observed for jump height (ηp^2^ = .102) and negative power (ηp^2^ = .106). A significant interaction effect between leg dominance and group was found for propulsive time. In propulsive time the pwMS showed an higher difference (ηp^2^ = .105) between the dominant and non-dominant leg compared to HC (ηp^2^ = .344).

Figure 3 shows the summary of rmANCOVA results on SLCMJ parameters with Bonferroni adjusted main effects analyses for leg dominance (dominant and non-dominant), group (pwMS-NM and HC), and group × leg dominance interactions and Bonferroni-based post-hoc comparisons for all jump parameters. Significant differences between pwMS-NM and HC were observed in flight time, force at zero velocity, negative and positive power, and jump height for both the dominant and non-dominant leg, with higher effect sizes for the non-dominant leg (see Figure 3). In FTCTR and braking time, the groups differed significantly only in the non-dominant leg. In addition, both groups showed almost similar peak force on both the dominant and the non-dominant leg. Furthermore, the jumping performance of the dominant and non-dominant leg differed significantly (*P* < .001) in all investigated jump parameters in pwMS-NM and HC, with higher effect sizes in pwMS-NM (see Figure 3).

### Correlation Between Jump Parameter and EDSS in pwMS-NM

Overall, the bivariate comparison of the jump parameters and clinical outcome score (EDSS) showed mild association (see supplement). The highest correlation coefficients were observed for positive power of the non-dominant (*r* = −.366, *P* < .001) and dominant leg (*r* = −.337, *P* < .001) in relation to the pyramidal FSS. The jump parameters of the non-dominant leg showed overall higher correlation coefficients with the EDSS and its FSS, than the dominant leg and the LSI. The parameter jump height was the only parameter that showed a significant correlation in all 3 categories (non-dominant leg, dominant leg, and LSI) with the pyramidal FS. Considering the relationship between limb symmetry and degree of disability, the highest correlation coefficient was found between LSI of flight time and cerebellar FSS (*P* = .001, *r* = .301), followed by positive power (*P* = .006, *r* = .256), an d FTCTR (*P* = .007, *r* = 0.256).

## Discussion

In this study, we objectively evaluated early lower limb neuromuscular deficits and asymmetries between pwMS and HC using SLCMJ on a force plate. To evaluate the appropriateness of the SLCMJ in detecting lower limb impairments at an early and subtle stage, we established a group of people with MS who possessed normal muscle strength (pwMS-NM), comparable to that of the HC group. Our main findings suggest that the SLCMJs, as a new and objective assessment, are suitable for identifying subclinical muscle weakness and asymmetries compared to the MMT.

The results of this study are consistent with our previous study and Kirkland et al, who indicated that jumps can detect and monitor subtle sensorimotor deficits in pwMS.^21,24,25^ In contrast to Kirkland et al we used SLCMJ on a force plate instead of horizontal jumps on a instrumented walkway. All other studies that investigated jumping in MS examined bipedal jumps.^21,23,24^ Our study is the first to characterise single-leg jumps in MS.Our results suggest that pwMS-NM have significantly reduced SLCMJ performance compared to HC with the highest effect sizes for negative power and jump height. These results are consistent with Stagsted et al who showed that pwMS often demonstrate a decrease in muscle strength and performance, especially during fast dynamic muscle contractions.^43^

Additionally, larger between-group effect sizes were observed for the non-dominant leg compared to the dominant leg. Peak force was the only jump parameter where the groups did not differ significantly on both legs. Eagles et al^35^ found in their study, that peak force values do not provide a valuable insight into movement performance because Newton’s second law (force = mass × acceleration) shows that greater impulses are not produced by simply increasing peak force, but rather by increasing the force at each moment of force application. The performance of SLCMJ in pwMS is characterized by longer contraction time, lower power in the eccentric and concentric phase, shorter flight time, and lower jump height. In both groups the concentric phase was longer than the eccentric phase and the maximum concentric force was greater than the maximum eccentric force.

The SLCMJs could be suitable for detecting early asymmetries between the leg sides of the lower limb in pwMS-NM. In both groups (pwMS-NM and HC), jumping performance differed significantly between the dominant and non-dominant leg. However, the pwMS-NM had a significantly higher effect size, indicating that the pwMS-NM had a more severe difference between the sides of the leg than the HC. These results suggest that pwMS not only have physiological differences but also subclinical asymmetries in neuromuscular function at an early stage of disability. A significant interaction effect between leg dominance and group was only found for propulsive time. These observations are consistent with the descriptive calculations of the LSI, where the pwMS-NM had a higher LSI of propulsive time compared to the HC (pwMS-NM: 16.5% vs HC: 12%). The pwMS-NM show a longer concentric phase on the non-dominant leg than the HC, while both groups did not differ on the dominant leg. This suggests a restricted coordinated activation of the muscles during the stretch-shortening cycle in MS, particularly on the non-dominant leg.

Our study demonstrated a small correlation between leg asymmetries and level of disability (EDSS) in pwMS. With the highest correlation coefficient, the LSI of flight time increased by ~16% with increasing impairment (FSS0-FSS2) of cerebellar FS. This indicates that the leg side difference is related to the clinical abnormalities in the different FS. These results are consistent with the findings of Farrell et al who found that strength asymmetry increases with increasing disability.^8^

We found that the 2 jump parameters, jump height and negative power, were especially suitable to show differences between pwMS and HC, as well as between legs. These results are consistent with our previous study, where we found that these 2 parameters could distinguish the CMJ performance between pwMS and HC, both with normal pyramidal, sensory, and cerebellar function.^24^

These ﬁndings have clinical relevance in the care of pwMS. Early identification of neuromuscular deficits and asymmetries is important for early rehabilitation to slow the progression of disability before measurable motor impairment occurs. Studies showed that early pharmacological therapy is effective.^44-46^ However, early neurorehabilitation in MS has received little attention in research and clinical practice. Riemenschneider et al show that exercise therapy is started at a relatively late stage of the disease when it is mainly used for symptomatic treatment.^47^ A “window of opportunity” in the early stages of MS where early neurorehabilitation can have a positive impact on functional and neurological reserve.

The SLCMJ as a functional assessment of muscle activity allows a better simulation of everyday movements as it is based on the principle of the stretch-shortening cycle that occurs during natural movements such as walking and running.^48^ Our results, combined with others, could mean that in the future, jumps could be used not only as a testing tool, but also as a useful tool in the treatment or rehabilitation of neurological disorders.^49^

Compared to the MMT, which only test the subjectively isolated concentric strength, the SLCMJ on a force plate measures objectively the functional activity of the entire lower limb. This use of objective outcome measures using technological resources represents a promising opportunity for MS specialists and could be the key to early detection of progression.^9^ Furthermore, this objective data can be used to develop a digital twin, a revolutionary tool to improve diagnosis, monitoring, and therapy refining patients’ well-being, saving economic costs, and enabling prevention of disease progression.^11^

Our study has the limitations of a cross-sectional study. Future studies should be longitudinal to determine whether the SLCMJ can be used as a standardized measure for the mid- and long-term assessment of disease progression and treatment response in MS. Additionally, the reliability and validity of the SLCMJ has only been investigated in athletes and not in pwMS. Future studies should investigate this in order to define measurement variability and cut-off scores.

This study used highest jump height to define the dominant leg. Previous studies have shown different methods to defined the dominant leg: in a self-determined way as the kicking leg,^50^ foot used for stair climbing,^28^ leg used to regain balance after an unexpected perturbation,^51^ weaker and stronger leg,^28^ and under consideration of dominance questionnaires. Future studies should investigate which definition is suitable to define the dominant leg for SLCMJs in MS.

In addition, future studies should use other objective measures, such as the isokinetic dynamometer or electromyographic measurement, in combination with the SLCMJ to assess muscle strength and asymmetry objectively. Furthermore, the Edinburgh Handedness Questionnaire should be used in relation to SLCMJ to investigate the association between hand dominance and leg dominance.

In this study, the most common method of calculating the LSI was used Parkinson et al reviewed various quantitative methods for calculating and interpreting force asymmetry, as well as limit values for defining asymmetry given in the literature.^6^ The results of this review showed inconsistent implementation in the quantification and interpretation of lower limb muscle strength asymmetry. In future studies, individual and sample-specific thresholds are needed to improve robust calculation methods for LSI and to establish appropriate limb asymmetry thresholds for different samples, especially for MS. Another limitation was that no additional functional tests were performed in relation to the SLCMJ. If subclinical asymmetry in MS can be detected, it would be interesting to investigate whether these are associated with early impairments in balance and walking ability. Another limitation was that no additional functional tests were performed in relation to the SLCMJ. Future studies should investigate the relationship between asymmetry using SLCMJ and functional tests such as balance and gait assessments.^52^

## Conclusion

Our study is the ﬁrst to provide evidence that the SLCMJ on a force plate, as a new assessment in MS, could be able to detect subclinical neuromuscular deficits and strength asymmetries of the lower limb in pwMS, who do not have strength deficits and asymmetries according to MMT. The SLCMJ on a force plate can add comprehensive objective and metric parameters, which provide important additional information about the pyramidal, cerebellar, and sensory functions of the EDSS. This comprehensive analysis of lower limb muscle function may be useful in advancing the necessary implementation of individualized innovative neurorehabilitation management of MS at an early stage.^11,53,54^ Future studies should investigate the SLCMJ in relation to other objective muscle function measures and other methods to define the dominant leg for SLCMJs in MS. This will provide evidence of the reliability and validity of the SLCMJ in pwMS.

## Supplemental Material

sj-docx-1-nnr-10.1177_15459683241245964 – Supplemental material for Sensitive Identification of Asymmetries and Neuromuscular Deficits in Lower Limb Function in Early Multiple Sclerosis Supplemental material, sj-docx-1-nnr-10.1177_15459683241245964 for Sensitive Identification of Asymmetries and Neuromuscular Deficits in Lower Limb Function in Early Multiple Sclerosis by Anne Geßner, Maximilian Hartmann, Anikó Vágó, Katrin Trentzsch, Dirk Schriefer, Jan Mehrholz and Tjalf Ziemssen in Neurorehabilitation and Neural Repair
