# Supplementary material for: Sensitive Identification of Asymmetries and Neuromuscular Deficits in Lower Limb Function in Early Multiple Sclerosis
Source: Neurorehabil Neural Repair. 2024 Apr 13;38(8):570–81. doi: 10.1177/15459683241245964 (PMC11308279; doi:10.1177/15459683241245964)
Supplement: sj-docx-1-nnr-10.1177_15459683241245964 – Supplemental material for Sensitive Identification of Asymmetries and Neuromuscular Deficits in Lower Limb Function in Early Multiple Sclerosis [file sj-docx-1-nnr-10.1177_15459683241245964.docx]

|  | **EDSS** | **Pyramidal FSS** | **Cerebellar**  **FSS** | **Sensory**  **FSS** | **EDSS** | **Pyramidal FSS** | **Cerebellar**  **FSS** | **Sensory**  **FSS** | **EDSS** | **Pyramidal FSS** | **Cerebellar**  **FSS** | **Sensory**  **FSS** |
| --- | --- | --- | --- | --- | --- | --- | --- | --- | --- | --- | --- | --- |
|  | **Non-Dominant Leg** | | | | **Dominant Leg** | | | | **LSI** | | | |
| **Temporal Parameter** | | | | | | | | | | | | |
| **FT** | - 0.207*  p = 0.028 | - 0.221*  p = 0.019 | - 0.200  p = 0.035 | - 0.095  p = 0.318 | - 0.138  p = 0.147 | - 0.099  p = 0.298 | 0.042*  p = 0.661 | - .214*  p = 0.023 | 0.171  p = 0.071 | 0.251  p = 0.008 | 0.301  p = 0.001 | - 0.140  p = 0.140 |
| **ET** | 0.311  p < 0.001* | 0.181  p = 0.056 | 0.169  p = 0.075 | 0.199  p = 0.036* | 0.153  p = 0.108 | 0.096  p = 0.316 | 0.119  p = 0.212 | 0.076  p = 0.423 | 0.206  p = 0.029* | 0.151  p = 0.112 | 0.081  p = 0.397 | 0.167  0.078 |
| **CT** | -0.010  p = 0.920 | 0.128  p = 0.180 | -0.049  p = 0.606 | -0.081  p = 0.397 | -0.099  p = 0.301 | 0.045  p = 0.640 | -0.095  p = 0.318 | -0.101  p = 0.288 | 0.053  p = 0.579 | 0.093  p = 0.330 | 0.110  p = 0.248 | -0.050  p = 0.598 |
| **Kinetic Parameter** | | | | | | | | | | | | |
| **FZV** | - 0.073  p = 0.444 | - 0.072  p = 0.449 | - 0.173  p = 0.069 | - 0.204  p = 0.031* | - 0.038  p = 0.691 | - 0.071  p = 0.460 | - 0.160  p = 0.091 | - 0.081  p = 0.397 | 0.091  p = 0.338 | 0.005  p = 0.955 | 0.020  p = 0.838 | 0.155  p = 0.104 |
| **PF** | 0.034  p = 0.719 | 0.091  p = 0.340 | -0.012  p = 0.898 | - 0.044  p = 0.648 | 0.036  p = 0.710 | 0.106  p = 0.265 | - 0.022  p = 0.815 | - 0.029  p = 0.759 | - 0.100  p = 0.293 | - 0.029  p = 0.765 | - 0.057  p = 0.552 | 0.025  p = 0.793 |
| **NP** | 0.097  p =0.309 | 0.203  p = 0.032* | 0.208  p =0.028* | 0.215  p =0.023* | 0.117  p = 0.219 | 0.216  p = 0.022* | 0.240  p = 0.011* | 0.189  p = 0.046* | 0.011  p = 0.911 | - l0.025  p = 0.794 | 0.042  p = 0.658 | 0.074  p = 0.438 |
| **PP** | - 0.278  p =0.003* | - 0.366  p < 0.001* | - 0.273  p =0.004* | - 0.194  p =0.041* | - 0.270  p = 0.004* | - 0.337  p < 0.001* | - 0.224  p =0.018* | - 0.196  p =0.038* | 0.131  p = 0.168 | 0.188  p = 0.047* | 0.256  p = 0.006* | 0.051  p = 0.591 |
| **Performance Parameter** | | | | | | | | | | | | |
| **SH** | - 0.233  p = 0.013* | - 0.311  p < 0.001* | - 0.235  p = 0.012* | - 0.118  p = 0.214* | - 0.168  p = 0.076* | - 0.246  p = 0.009* | - 0.150  p = 0.114 | - 0.115  p = 0.229 | 0.175  p = 0.064 | 0.188  p = 0.047* | 0.218  p = 0.021* | - 0.059  p = 0.534 |
| **FTCTR** | - 0.110  p = 0.250 | - 0.156  p = 0.100 | - 0.208  p = 0.028* | - 0.178  p = 0.061 | - 0.037  p = 0.701 | - 0.057  p = 0.550 | - 0.009  p = 0.922 | - 0.067  p = 0.482 | 0.127  p = 0.181 | 0.174  p = 0.066 | 0.256  p = 0.007* | 0.210  p = 0.026* |

Supplement 1. Correlation between jump parameters and EDSS including pyramidal, cerebellar and sensory FSS in 112 pwMS according to Spearman.

Abbreviations: FSS = functional system score
